# In Vitro and Ex Vivo Evaluation of Penetratin as a Non-invasive Permeation Enhancer in the Penetration of Salmon Calcitonin through TR146 Buccal Cells and Porcine Buccal Tissues

**DOI:** 10.3390/ph13110408

**Published:** 2020-11-21

**Authors:** Taekwang Keum, Gyubin Noh, Jo-Eun Seo, Santosh Bashyal, Sangkil Lee

**Affiliations:** 1 College of Pharmacy, Keimyung University, 1095 Dalgubeol-daero, Dalseo-gu, Daegu 42601, Korea; gtk02@daum.net (T.K.); rhgyubin@naver.com (G.N.); joeun0405@hanmail.net (J.-E.S.); bashyal.santosh18@gmail.com (S.B.); 2 Center for Forensic Pharmaceutical Science, 1095 Dalgubeol-daero, Dalseo-gu, Daegu 42601, Korea

**Keywords:** cell penetrating peptide, Penetratin, salmon calcitonin, buccal drug delivery, TR146 cells

## Abstract

Buccal tissues are considered one of the potential alternative delivery route because of fast drug absorption and onset of action due to high vascularization and a non-keratinized epithelial membrane. In this study, the effect of Penetratin on the permeation of salmon calcitonin (sCT), a model macromolecular peptide drug, through TR146 buccal cells and porcine buccal tissues has been evaluated. To observe permeation profile of sCT, TR146 buccal cells were treated with Alexa 647 conjugated sCT (Alexa 647-sCT) with different concentrations of fluorescein isothiocyanate -labeled Penetratin (FITC-Penetratin) ranging from 0 to 40 μM, and analyzed using flow cytometry and confocal laser scanning microscopy. Intracellular penetration of FITC-Penetratin rapidly increased at low concentrations from 0 to 15 μM and it gradually increased at concentrations above 15 μM. Intracellular penetration of Alexa 647-sCT enhanced with the increase of FITC-Penetratin concentration. When TR146 cell layers and buccal tissues were co-treated with sCT and Penetratin as permeation enhancer, the flux of sCT increased as per Penetratin concentration. Compared to the control, 12.2 μM of Penetratin enhanced the flux of sCT in TR146 cell layers and buccal tissues by 5.5-fold and 93.7-fold, respectively. These results strongly suggest that Penetratin may successfully act as a non-invasive permeation enhancer for macromolecular peptide drug delivery through buccal routes.

## 1. Introduction

The biopharmaceuticals market has increased significantly with the development of biotechnology in the past decades, and researches on formulations and drug delivery via various administration routes are actively being conducted [1]. Among various delivery routes, oral administration is often preferred, but the oral route is not suitable for protein drugs due to the hepatic first pass effect, hydrolysis in the gastrointestinal tract, and enzymatic degradation in the small intestine [2,3]. To overcome these hurdles, protein drugs are administered through subcutaneous or intramuscular routes, generally with multiple injections because of their short half-life [4]. However, these invasive routes have low patient compliance because of pain, needle phobia, phlebitis, and tissue necrosis induced by frequent injections [5]. Therefore, alternative drug delivery routes through the vagina, rectum, skin, nasal, buccal, and pulmonary organs have been investigated [6,7,8]. Among these alternative routes, the buccal route is considered a suitable systemic delivery of peptide and protein drugs because of the high vascularization and non-keratinized buccal epithelium [9,10,11,12]. Buccal administration avoids the first pass effect and enzymatic metabolism, and it also allows a short turnover time and ease of administration by avoiding needles [13]. To date, the delivery of protein drugs, such as secretin, substance P, insulin, calcitonin, and growth hormone, through buccal administration has been studied [4]. However, buccal delivery of biopharmaceuticals is still a major challenge due to the presence of a permeability barrier the buccal mucosa [9]. Since the permeability of the buccal mucous membrane is low, absorption enhancers are required [9]. Various physical techniques and chemical enhancers are used to enhance the permeability of these biopharmaceuticals across the buccal mucosa. However, there are limitations to the use of physical techniques, such as iontophoresis and sonication because of their inaccessibility at home and high cost, and chemical enhancers are toxic at high concentrations [14].

Cell penetrating peptides (CPPs) were considered as attractive permeation enhancers that could be used to enhance the delivery of protein drugs across the biomembrane. Tat, which was discovered in 1988, was the first known CPP, followed by the homeodomain of antennapedia in 1991. The specific residue of CPPs were subsequently recognized as an important factor affecting cell permeability [13]. CPPs are relatively short (less than 30 amino acid residues), cationic, and amphipathic peptides capable of aiding the penetration of macromolecules through the cell membrane with low cytotoxicity [15,16,17]. Tréhin et al. revealed the cellular uptake of human calcitonin derived CPPs, Tat and Penetratin in various cell lines, such as MDCK, Calu-3, and TR146 cell. Among them, Penetratin showed the highest cellular uptake by TR146, a buccal cell line [18].

In recent studies, CPPs have been used to increase the efficiency of drug delivery. From the perspective of covalent bonding, insulin each conjugated with R9, K9, and Tat was delivered to the lungs, and insulin conjugated with LMWP(low molecular weight protamine) and Tat was administered orally [19,20,21,22]. From the perspective of non covalent bonding, Penetratin is actively studied. l,d-Penetratin were used by simple mixing formulation to enhance permeation of insulin through Caco-2 cells [23]. In oral and nasal delivery study, insulin absorption was enhanced by l,d-Penetratin [24,25]. Penetratin enhanced permeation of glucagon-like peptide-1 and exendin-4 in nasal and intestinal delivery. Permeation of interferon-β co-administrated with Penetratin increase in nasal delivery [26]. The use of CPPs as a permeation enhancer has been ongoing for oral, lung, nasal, and topical administration. However, there has been little study on its use for buccal administration [16,27,28,29].

The main aim of the present study was to investigate the concentration of Penetratin on the permeation of macromolecular drugs. We selected salmon calcitonin (sCT) as a model peptide drug and Penetratin as a CPPs candidate. Fluorescein isothiocyanate -labeled Penetratin (FITC-Penetratin) and Alexa Fluor 647-conjguated sCT (Alexa 647-sCT) were used in TR146 human buccal cells and porcine buccal tissues. In vitro cellular uptake of FITC-Penetratin was checked using flow cytometry and confocal laser microscopy. It was confirmed, through a cell uptake study, whether the effect of enhancing sCT penetration of penetratin appears in energy-abolishing conditions. The permeation and distribution of sCT was evaluated in TR 146 cells and porcine buccal tissue to confirm the permeation enhancing effect of Penetratin.

## 2. Results

### 2.1. Cytotoxicity

**Figure 1:**
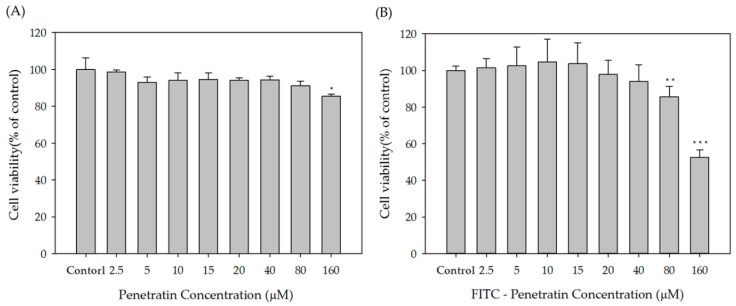
Cell viability of TR146 cells following 24 h of incubation with various concentrations of Penetratin (**A**) and FITC-Penetratin (**B**). Error bars represent SD (*n* = 5). * *p* < 0.05 versus control, ** *p* < 0.01 versus control and *** *p* < 0.001 versus control.

To determine the optimal concentration of Penetratin and FITC-Penetratin, a cytotoxicity study was performed using the TR146 human buccal cell line. The concentration of Penetratin and FITC-Penetratin treated to cells ranged from 2.5–160 μM. Penetratin did not induce cytotoxicity below 160 μM (Figure 1A) and FITC-Penetratin did not induce cytotoxicity below 80 μM (Figure 1B). Therefore, cellular uptake and permeation studies were conducted in the rage of 0–40 μM where both Penetratin and FITC-Penetratin did not induce cytotoxicity.

### 2.2. FITC-Penetratin and Alexa 647-sCT Internalization into TR146 Cell

**Figure 2:**
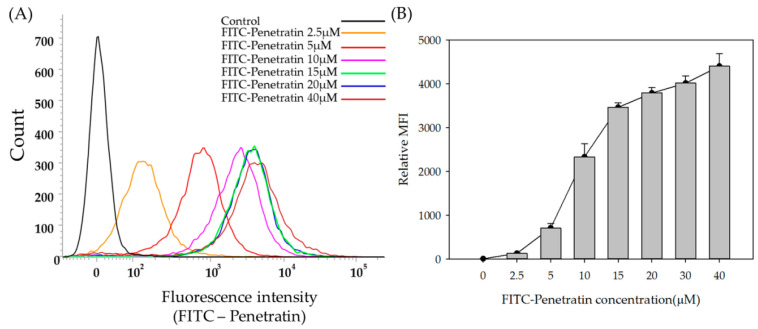
FACS data of FITC-Penetratin internalization in TR146 cells. Representative fluorescence intensity (**A**) and relative MFI values of FITC-Penetratin (**B**). All data were represented by mean ± S.D (*n* = 3).

**Figure 3:**
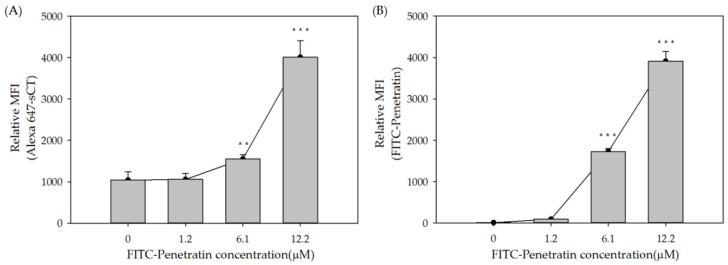
FACS data of FITC-Penetratin and Alexa 647-sCT internalization in TR146 cells. Relative MFI values of FITC-Penetratin (**A**) and relative MFI values of Alexa 647-sCT (**B**). All data were represented by mean ± S.D (*n* = 3). ** *p* < 0.01 versus control and *** *p* < 0.001 versus control.

**Figure 4:**
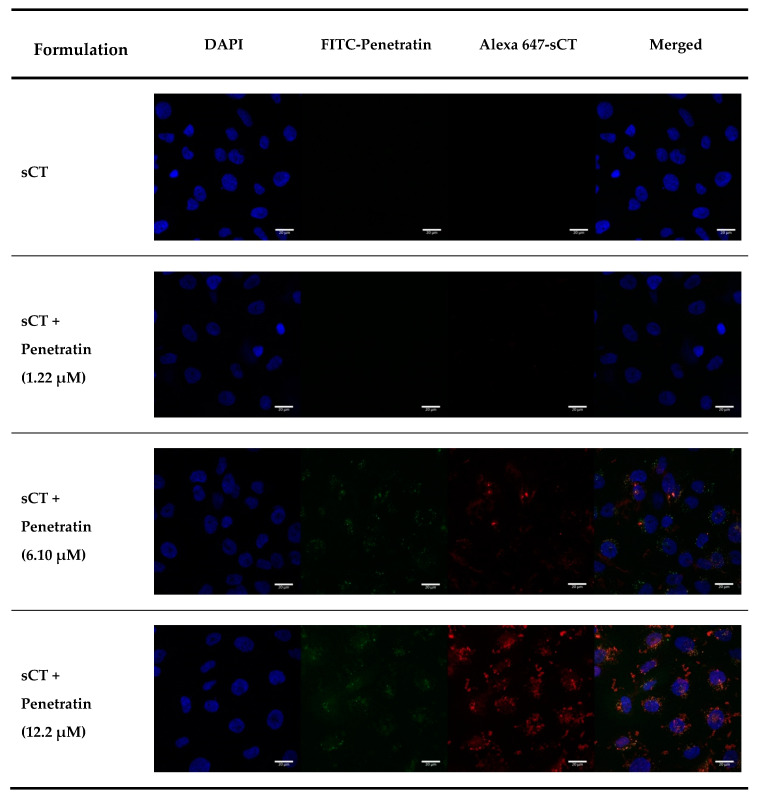
CLSM images of FITC-Penetratin and Alexa 647-sCT internalization in TR146 cells after 2 h of incubation at 37 °C. Notes: Blue: nuclei stained with DAPI; green: FITC-Penetratin; red: Aelxa 647-sCT, respectively.

To study the intracellular penetration and permeation enhancing effect of Penetratin, FITC-Penetratin and Alexa 647-sCT were used and analyzed by Flow cytometry (FACS) and confocal laser scanning microscopy (CLSM) at 37 °C. TR146 cells were seeded and incubated for 24 h, and cells were treated with various concentrations of FITC-Penetratin for 2 h. As the concentration of FITC-Penetratin increased, the relative mean fluorescence intensity (MFI) value also increased (Figure 2A). The relative MFI increased steeply from 2.5 to 15 μM, but increased more gradually from 15 to 40 μM (Figure 2B). Based on these results, we evaluated the effect of Penetratin on the permeation of sCT. In a different set of experiments, the relative MFI of FITC-Penetratin increased depending on the concentration of Penetratin (Figure 3A) and that of Alexa 647-sCT increased from 6.1 μM (Figure 3B). Qualitative cellular uptake imaging study performed by confocal microscopy showed that the intracellular uptake of FITC-Penetratin and Alexa 647-sCT increased as the FITC-Penetratin concentration was increased (Figure 4).

**Figure 5:**
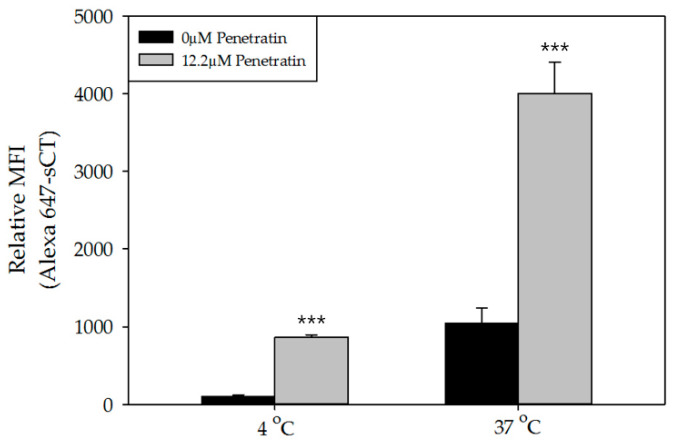
FACS data of FITC-Penetratin and Alexa 647-sCT internalization in TR146 cells. Relative MFI value of Alexa 647-sCT from the group each treated with 0 and 12.2 μM of Penetratin at 4 °C and 37 °C. All data were represented by mean ± S.D (*n* = 3). *** *p* < 0.001 versus control.

To understand energy-independent internalization on the permeation enhancing effect of Penetratin, 12.2 μM of FITC-Penetratin and 40 μg of Alexa 647-sCT was analyzed through FACS at 4 °C. Internalization of FITC-Penetratin was confirmed at 4 °C. Relative MFI of Alexa 647-sCT increased by 8.45-fold in comparison with the control at 4 °C (Figure 5).

### 2.3. In Vitro Cell Permeation Study

**Figure 6:**
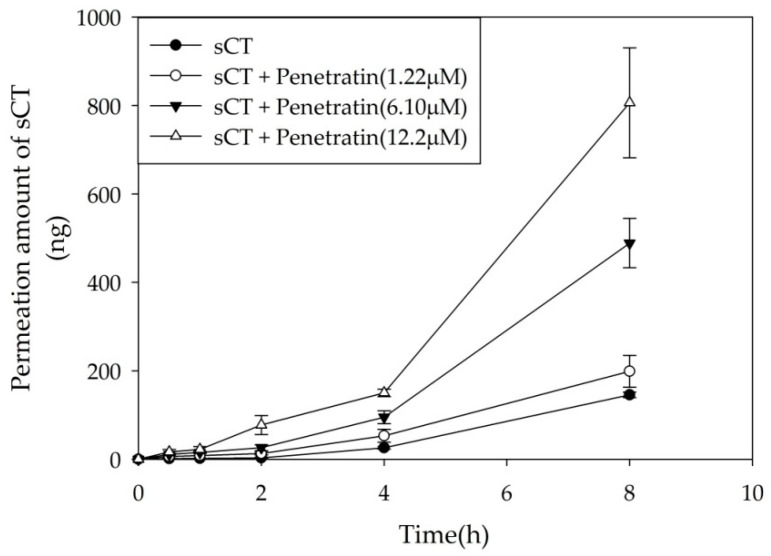
sCT permeation across TR146 cell layer. Cellular permeation of sCT increased as Penetratin concentration dependently. All data were represented by mean ± S.D (*n* = 3).

Based on the cellular uptake study, the permeation enhancing effect of Penetratin on cellular permeation of sCT was evaluated using Transwell. The permeation of sCT increased as the concentration of Penetratin increased (Figure 6). In comparison to the control, 12.2 μM of Penetratin increased the *J_s_* value of sCT by 5.5-fold (Table 1). Transepithelial electrical resistance (TEER) values were measured to confirm the cellular viability before and after the study. TEER of all experiment groups maintained 90% of the original value of fresh cell membranes (Table 2).

### 2.4. Ex Vivo Buccal Tissues Permeation

**Figure 7:**
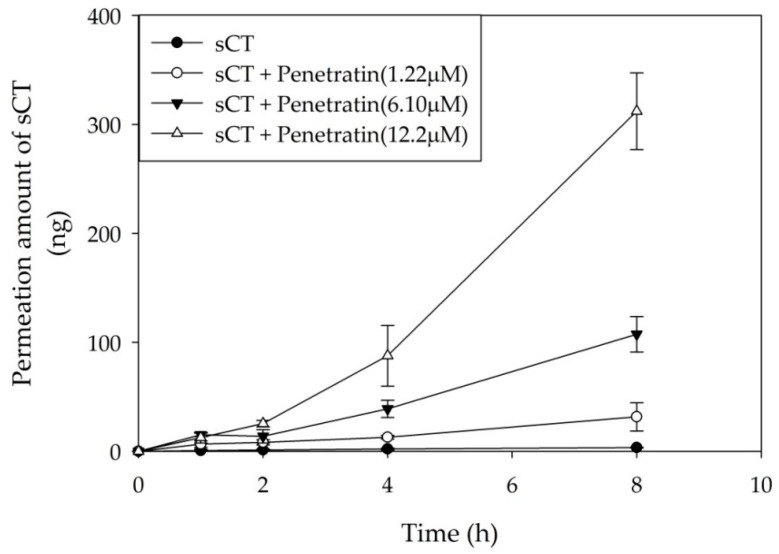
Permeation profile of sCT across porcine buccal tissues. All data were represented by mean ± S.D (*n* = 3).

To investigate the absorption enhancing effect of Penetratin, porcine buccal tissues were used in a permeation study. Forty micrograms of sCT were mixed with various concentrations of Penetratin, and applied to buccal tissues for 8 h. Compared to the control, permeation of sCT increased in a Penetratin concentration-dependent manner (Figure 7) and the permeated amount of sCT increased 93.7-fold (Table 3). The porcine buccal tissue permeation parameters are summarized in Table 3.

### 2.5. CLSM Study Using Alexa 647-sCT and FITC-Penetratin in Buccal Tissues

**Figure 8:**
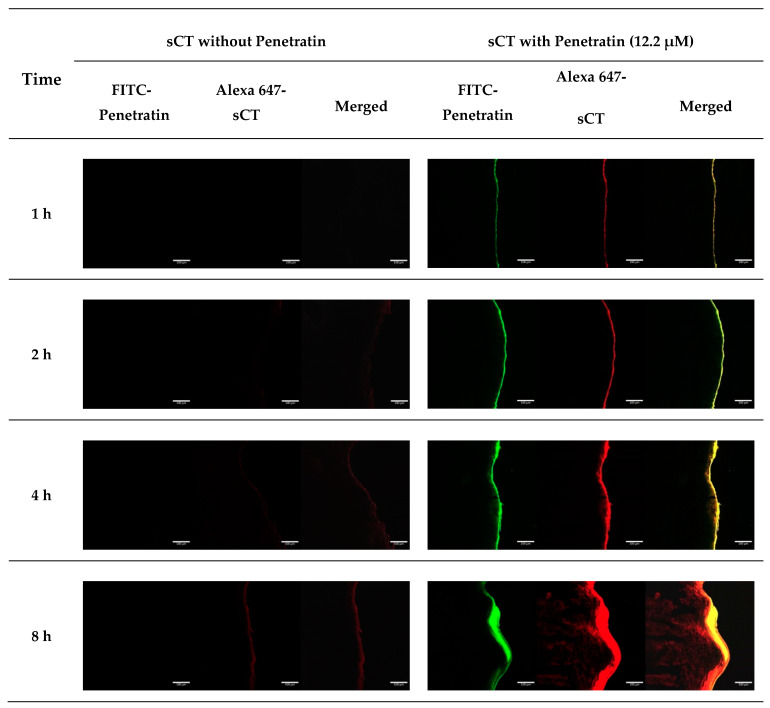
CLSM images of FITC-Penetratin and Alexa 647-sCT internalization in buccal tissues at 37 °C. Notes: Green: FITC-Penetratin; red: Alexa 647-sCT.

Confocal microscopy image analysis was used to confirm the permeating enhancing effect of Penetratin on Alexa 647-sCT. Enhanced permeation of Alexa 647-sCT was seen with 12.2 μM of Penetratin. The fluorescence intensity was stronger from 1 h to 8 h. However, no enhancing effect was observed without Penetratin (Figure 8).

## 3. Discussion

CPPs can penetrate cell membranes, and many studies have used CPPs for the delivery of biopharmaceuticals for the treatment of viruses, bacterial infections and cancers [30]. CPPs act as absorption enhancers through covalent bonding to drugs or drug delivery carriers or non-covalent physical mixing [31,32]. CPPs can be covalently conjugated to various cargo, such as liposomes, polymeric nanoparticles, proteins, peptides, and DNA by chemical cross-linking, and showed low toxicity and high drug delivery efficiency in vitro and in vivo [17]. Physical mixing of CPPs with drug molecules also promote cellular and tissue permeation of protein drugs [33,34,35].

Penetratin is one of the most promising CPPs. Penetratin has positively charged amino acids such as lysine and arginine, and hydrophobic amino acids. Various cargoes were delivered by overcoming the barrier of the cell membrane, but the mechanism of CPPs and Cargo delivery through the cell membrane is still unclear [36]. There is a possible explanation. At pH 7.4, where permeation and cell uptake experiments were conducted, sCT with a pI of 10.4 and penetratin have a positive charge, so there is no ionic interaction between penetratin and sCT. Penetratin and sCT form a hydrophobic interaction. The guanidium group of arginine contributes to binding to the cell membrane. After binding to the cell membrane, Penetratin permeate cell membrane due to the hydrophobic effect, which is the main force delivered into the cell [37].

As shown in Figure 1 and Figure 2, there was a rapid internalization of Penetratin from 15 μM to 160 μM with low cytotoxicity, suggesting that Penetratin can be used safely as a permeation enhancer. The relative MFI value of Penetratin rapidly increased up to 15μM but increased more gradually above 15 μM. This indicates that internalization of Penetratin increases according to its concentration. However, the amount of efflux also increases above a specific concentration. FACS and CLSM results showed that absorption of Penetratin enhanced Alexa 647-sCT increased along with concentration of Penetratin (Figure 3 and Figure 4). From the results of cellular uptake using CLSM, it was confirmed that fluorescence of FITC-Penetratin and Alexa 647-sCT overlapped and did not overlap when 6.10uM of FITC-Penetratin was treated. The presence of non-overlapping parts is that FITC-Penetratin did not promote cell uptake of Alexa 647-sCT. On the other hand, when 12.2uM of FITC-Penetratin was treated, most of FITC-Penetratin and Alexa 647-sCT overlapped. Permeation of sCT is promoted when there is an interaction between sCT and Penetratin.

The mechanisms of CPPs are under investigation by many researchers. Dom et al. reported that when COS-7 cells were treated with both, Penetratin (32 μM) and DNA at 4°C and 37 °C, the DNA was successfully internalized. However, when mutant Penetratin (in which tryptophan was substituted by phenylalanine) was used, DNA was internalized only at 37 °C and not at 4°C. These results show that the tryptophan in the Penetratin molecule plays an important role in drug delivery and uses energy independent internalization for the delivery of drugs [38]. In the present study, permeation of sCT through TR146 cells at energy-abolishing condition (4 °C) and biological condition (37 °C) was enhanced by 12.2 μM of Penetratin (Figure 5). Accordingly, we can suggest that the tryptophan in Penetratin plays an important role in the function of Penetratin as a permeation enhancer for sCT and energy independent internalization is related when penetratin promotes permeation of sCT in the TR146 buccal cell line. Additionally, the permeation enhancing effect of Penetratin is related to the energy independent internalization mechanism into cells.

TEER value represents the integrity and the function of tight junctions between cell layers. If a tight junction of cell layer loosen, membrane integrity also diminishes and penetration to the paracellular route increases [39]. The maximum TEER value of the TR146 cell layer incubated for 30 days in the in vitro cell permeation study was 71.30 Ω·cm^2^ [39,40]. When the TR146 cell layer was treated with Penetratin, TEER value did not differ significantly [18,41]. This confirmed that Penetratin does not have an impact on the function of tight junctions and indicates that Penetratin is not delivered via a paracellular route. TEER value did not change, however, the intracellular permeation of Alexa 647-sCT increased in a Penetratin concentration increases. Therefore, from the FACS, CLSM, and cell permeation studies using TR146 human buccal cells, we can deduce that the permeation enhancing effect of Penetratin happens through a transcellular route. As shown in Figure 7 and Figure 8, permeation of Alexa 647-sCT increased in a Penetratin concentration-dependent manner in porcine buccal tissues. As the concentration of penetratin increased, the amount of permeation increased in TR146 cells and buccal tissues, and the amount of permeation increased in cells compared to tissues. The reason for the difference in permeation amount is that tissues are multi-layered compared to cells. In permeation experiments in cells and tissues, Penetratin can permeate not only cell layers but also multi-layered tissues.

In the CLSM results, 12.2 μM of Penetratin showed increased fluorescence intensity in the tissues as time passed. Fluorescence of FITC-Penetratin and Alexa-647 sCT overlapped in the group treated with 12.2uM of FITC-Penetratin. Therefore, sCT was permeated by penetratin in porcine buccal tissues.

## 4. Materials and Methods

### 4.1. Materials

Salmon calcitonin was purchased from Bachem AG (Bubendorf, Switzerland). Penetratin (RQIKIWFQNRRMKWKK) and FITC labeled Penetratin (FITC-Penetratin) were purchased from Peptron Co., Ltd. (Daejeon, Korea). Alexa Fluor 647 NHS Ester, SnakeSkin^TM^ dialysis tubing (3.5 K MWCO), Fetal bovine serum (FBS), 0.25% trypsin-EDTA and penicillin/streptomycin were purchased from Thermo Fisher Scientific (Waltham, MA, USA). The sCT ELISA kit was purchased from Phoenix Pharmaceuticals (Burlingame, CA, USA). F-12 Nutrient mixture Ham (Ham’s F-12) was purchased from Welgene (Gyeongsan-si, Korea). The CellTiter 96^®^ Aqueous One Solution Cell Proliferation Assay kit (MTS) was purchased from Promega (Madison, WI, USA). All other chemicals and solvents were reagent grade.

### 4.2. Methods

#### 4.2.1. TR146 Cell Culture

The TR146 cell line (ECACC 10032305) was purchased from Public Health England (London, UK). TR146 cells were cultured in Ham’s F-12 supplemented with 10% FBS, 2 mM glutamine, penicillin (10,000 units/mL), and streptomycin (10,000 μg/mL), and incubated at 37 °C in 5% CO_2_. The media were replaced every 2–3 days. At 70–80% confluency, cells were split using 0.25% trypsin–EDTA.

#### 4.2.2. Cytotoxicity Assay

For the cytotoxicity assays of FITC-Penetratin and Penetratin, TR146 cells were seeded in a 96-well culture plate at a density of 1.0 × 10^4^ cells per well and incubated for 24 h at 37 °C in 5% CO_2_. After 24 h, the medium in each well was discarded, and 100 μL of FITC-Penetratin or Penetratin at 2.5, 5, 10, 15, 20, 40, 80, and 160 μM was added. After 24 h, cell viability was determined using an MTS assay.

#### 4.2.3. FITC-Penetratin and Alexa 647-sCT Internalization in TR146 Cell

For the internalization of FITC-Penetratin and Alexa 647-sCT, Alexa 647-sCT was synthesized. Alexa-647 and sCT were left at room temperature for 2 h to react, and the reaction was terminated using ammonium chloride. Purification of Alexa 647-sCT was done through dialysis.

TR146 cells were seeded in a 6-well culture plate and confocal dish at a density of 5.0 × 10^5^ cells per well, and incubated for 24 h at 37 °C in 5 % CO_2_. To determine the concentration at which optimal intracellular uptake of Penetratin occurs, FITC-Penetratin at 2.5, 5, 10, 15, 20, 30, and 40 μM were added and incubated for 2 h.

To confirm the effect of Penetratin on intracellular uptake of sCT, 1.22, 6.10 and 12.2 μM of FITC-Penetratin were added to 40 μg of Alexa 647-sCT and incubated for 2 h at 37 °C. For the energy-independent internalization of Penetratin, 40 μg of Alexa 647-sCT and 12.2 μM of FITC-Penetratin were added and incubated for 2 h at 4 °C (energy-abolishing condition) and 37 °C (biological condition). After 2 h, imaging study with flow cytometry (BD FACSverse; Becton Dickinson, Heidelberg, Germany) and confocal laser scanning microscopy (LSM 800, Zeiss, Oberkochen, Germany) were performed.

##### Flow Cytometry

TR146 cells were treated with a pre-determined amount of FITC-Penetratin and Alexa 647-sCT in a 6-well culture plate. They were washed twice with Phosphate buffer saline (PBS) and treated with 0.25% trypsin for 10 min to detach the cells. After collected, they were suspended in PBS and flow cytometry was immediately performed.

##### Confocal Laser Scanning Microscopy

The qualitative cellular uptake study of FITC-Penetratin was analyzed using CLSM. TR146 cells were treated with 40 μg of Alexa 647-sCT and 1.22, 6.10 and 12.2 μM of FITC-Penetratin in confocal dishes. They were washed three times with PBS, and 4% formalin was added for fixation in 10 min. After DAPI (4′, 6-diamidino-2-phenylindole) staining, the cells were observed immediately by CLSM.

#### 4.2.4. In Vitro Cell Permeation Study

TR146 cell was seeded in a 12-well Transwell insert (Corning Inc., Corning, NY, USA) at a density of 5 × 10^4^ cells per cm^2^, and the medium was changed every 2 days for 28–30 days. The experiment was conducted from apical to basolateral and HBSS-HEPES buffer was used as media. Forty micrograms of sCT and 1.22, 6.10, and 12.2 μM of Penetratin were added to apical chamber and kept at 37 °C. 500 μL of sampling was obtained at 0.5, 1, 2, 4 and 8 h. Transepithelial electrical resistance (TEER) value was measured before and after the experiment. TEER was calculated with the following Equation (1), where R_insert with cell_, R_insert without cell_, and A were insert filter areas. The permeated amount of sCT was analyzed using an sCT ELISA kit (Phoenix Pharmaceuticals, Burlingame, CA, USA):

TEER = (R_insert with cell_ − R_insert without cell_) × A (1)

#### 4.2.5. Pretreatment of Porcine Buccal Tissues

Buccal pre-treatment was performed according to the experimental method of Oh et al. [42]. Briefly, porcine buccal tissues were immediately collected after pigs were sacrificed from a local slaughterhouse. Adipose and connective tissues were removed from buccal mucosa using surgical scissors. To obtain buccal epithelium, tissues were put in pH 7.4 PBS at 60 °C for 1 min. They were evaluated in the following in vitro permeation study.

#### 4.2.6. Ex Vivo Buccal Tissues Permeation

Buccal permeability of sCT was evaluated using the Franz diffusion cell. The permeation area was 2.0 cm^2^, and the volume of receptor media was 12.5 mL. The receptor was filled with pH 7.4 PBS and stirred at 600 rpm at 37 °C. The Franz diffusion cell was equilibrated for 30 min after mounting the buccal epithelium. Forty micrograms of sCT and of 1.22, 6.10 and 12.2 μM of Penetratin, of which molecular ratios were 1:0.1, 1:0.5 and 1:1 in donor part, respectively, were added to the Franz diffusion cell. Half of a milliliter of sample was obtained at different time intervals (1, 2, 4, and 8 h) from the receptor part and analyzed by a sCT ELISA kit (Phoenix Pharmaceuticals, Burlingame, CA, USA).

#### 4.2.7. Permeation Parameters

The flux (*J_s_*) was calculated using the following Equation (2), where t (h) was permeation time, A($cm^{2}$) was permeation area, and $Q_{r}$(ng) was the permeated amount of sCT,

(2) $$J_{s}=\frac{Q_{r}}{A\cdot t}\left(\mathrm{ng}\cdot {\mathrm{cm}}^{-2}\cdot h^{-1}\right)$$

$K_{p}$ was calculated with the following Equation (3),

(3) $$K_{p}=\frac{J_{s}}{C_{d}}\left(\mathrm{cm}\cdot h^{-2}\right)\;$$

where *J_s_* was the flux from the steady state (ng⋅cm^−2^⋅h^−1^) and C_d_ was the initial concentration in the donor chamber (ng⋅cm^−3^). Finally, the enhancement ratio (ER) was obtained by dividing the *K_p_* value of each formulation with that of the control.

#### 4.2.8. CLSM Study Using Alexa 647-sCT and FITC-Penetratin in Buccal Tissues

Buccal tissue permeation was studied using 12.2 μM of FITC-Penetratin and 40 μg of Alexa 647-sCT. The buccal tissues obtained at different time intervals (1, 2, 4, and 8 h) were separated by a Franz Diffusion Cell. The separated tissues were fixed with OCT compound and frozen in a liquid nitrogen freezer. They were sliced in 7 μm using a cryostat (Cryotome FE, Thermo Fisher Scientific, Waltham, MA, USA) and were then fixed onto a slide and the cover was attached.

Buccal tissue permeation of Alexa-647 sCT and FITC-Penetratin was evaluated by CLSM.

#### 4.2.9. Statistical Analysis

Statistical analysis was performed by one-way ANOVA test. Data are presented as mean ± standard deviation (SD). The Holm-Sidak method was used for multiple comparison procedures. For a *p* value of less than 0.05, 0.01, or 0.001, single, double, or triple asterisks were used, respectively.

## 5. Conclusions

In this study, the permeation enhancing effect of Penetratin was investigated using sCT, a model macromolecular drug, in TR146 buccal cells and porcine buccal tissues. In the cytotoxicity test, Penetratin showed low cytotoxicity in TR146 cells and was considered safe at experimental concentrations. From the cell internalization study under different temperatures, the permeation enhancing mechanism of Penetratin was found to be an energy independent phenomenon. The Transwell and porcine buccal tissue permeation study revealed that a physical mixture of sCT with Penetratin was effective in buccal delivery of sCT. We can conclude that Penetratin may successfully be used as buccal delivery enhancer for proteins and peptides.

## Figures and Tables

**Table 1:** Permeation parameters calculated from the TR146 cell permeation study.

sCT (μg)	Concentration of Penetratin (μM)	*J_s_* (ng·cm^−2^·h^−1^)	*K_p_* (cm·h^−2^) × 10^−3^	ER
40	0	16.091 ± 0.560	0.201 ± 0.007	1.0
	1.22	22.004 ± 3.247	0.275 ± 0.041	1.4
	6.10	54.075 ± 5.053	0.676 ± 0.063	3.4
	12.2	89.190 ± 11.227	1.115 ± 0.140	5.5

**Abbreviations**: *J_s_*, Flux; *K_p_*, Permeability coefficient; ER, Enhancement ratio.

**Table 2:** TEER value for formulations before and after permeability experiments through TR146 cell layers.

sCT (μg)	Concentration of Penetratin (μM)	$\mathrm{TEER}\;\mathrm{Value}\;(\Omega \cdot {\mathrm{cm}}^{2})$		Recovery (%)
		Before	After	
40	0	62.72 ± 5.20	60.48 ± 5.88	96.31 ± 1.78
	1.22	63.47 ± 3.99	59.73 ± 2.94	94.21 ± 1.90
	6.10	61.23 ± 6.00	60.11 ± 6.00	98.17 ± 2.64
	12.2	63.84 ± 6.42	61.60 ± 6.62	96.52 ± 4.97

**Table 3:** Permeation parameters calculated from the sCT transbuccal permeation study.

sCT (μg)	Concentration of Penetratin (μM)	*J_s_* (ng·cm^−2^·h^−1^)	*K_p_* (cm·h^−2^) × 10^−^^3^	ER
40	0	0.208 ± 0.018	0.005 ± 0.000	1.0
	1.22	1.973 ± 0.660	0.049 ± 0.017	9.5
	6.10	6.707 ± 0.832	0.168 ± 0.021	32.2
	12.2	19.507 ± 1.794	0.488 ± 0.045	93.7

**Abbreviations**: *J_s_*, Flux; *K_p_*, Permeability coefficient; ER, Enhancement ratio.
